# Meeting Abstracts from the British Society of Breast Radiology annual scientific meeting 2023

**DOI:** 10.1186/s13058-024-01786-w

**Published:** 2024-04-17

**Proffered Papers**

**Monday 6 November 2023**

**1:10 PM–2:00 PM**

*Abstracts listed in presenting order*

## O1. Recovery of Screening Mammography Volumes Following the Onset of the COVID-19 Pandemic: An International Multi-Center Study

### Dr Jean Seely^1^, Prof Mohamed Abdolell^2,3^, Dr Jennifer Payne^2,3^, Dr Sian Iles^2,3^, Dr Georgia Spear^4^, **Dr Nisha Sharma**^5^, Dr Laurie R. Margolies^6^, Dr Sylvia Heywang-Köbrunner^7^, Dr Toni Vomweg^8^

#### ^1^The Ottawa Hospital, Ottawa, Canada; ^2^Nova Scotia Health Authority, Canada; ^3^Dalhousie University, Halifax, Canada; ^4^NorthShore HealthSystem, Evanston, United States; ^5^Leeds Teaching Hospital, NHS Trust, Leeds, United Kingdom; ^6^Mount Sinai Health System, New York, United States; ^7^Referenzzentrum Mammographie Munchen, Munich, Germany; ^8^Radiologisches Institut Dr von Essen Koblenz, Koblenz, Germany

*Breast Cancer Research* 2024, **26(1)**:O1

The COVID-19 pandemic led to recommendations for decreased or ceased breast screening services. Prior studies that examine the COVID-19 impact on mammography volumes are limited to single regions and rates of less than 9 months post-pandemic. This study aims to assess the recovery of screening mammography volumes from 3 months pre COVID-onset through 12 months post COVID-onset across 7 participating sites from four countries.

We collected mammography volume data from 7 breast screening services (Canada, Germany, USA, UK) between December 2019 and April 2021 using an artificial intelligence software tool. The study was approved by the research ethics at participating sites.

249,817 screening mammograms were collected. Of the 7 participating sites, 4 returned to and occasionally exceeded pre-COVID volumes, whereas the other 3 sites approached but did not fully return to pre-COVID volumes in the 12 months post COVID. Sites varied substantially in the time taken to re-initiate screening mammography after the first COVID wave (1–6 months). One site shut down screening services in successive COVID waves, while other sites experienced short dips in volumes but generally recovered within one month.

In the 1-year period post COVID-onset, mammography screening volumes recovered to varying extents at differing rates, with some sites returning to pre-pandemic levels and others lagging behind. This international multi-center study may inform future opportunities for collaboration between sites to develop strategies for increasing screening volumes and sharing best practices for pandemic recovery.

## O2. Assessing the value of mammography in symptomatic patients with normal breast examination: A retrospective observational study at the Beaumont Breast Centre

### **Dr Matthew Common**^1,2^, Dr Pat Rohan^1,2^, Dr Cathal McCarthy^1,2^, Eithne Downey^1^, Dr Niamh Hambly^1^, Dr Neasa ní Mhuircheartaigh^1^, Dr Jennifer Kerr^1^, Prof. Colm Power^1,2^, Prof. Arnold Hill^1,2^, Dr Deirdre Duke^1,2^

#### ^1^Beaumont Breast Centre, Dublin, Ireland; ^2^Royal College of Surgeons in Ireland, Dublin, Ireland

*Breast Cancer Research* 2024, **26(1)**:O2

**Background:** Symptomatic breast centre attendances are increasing exponentially. According to national guidelines, all patients ≥ 35yrs receive mammography regardless of clinical exam, presenting complaint, or risk profile.

**Purpose:** This study examines the use of breast imaging in symptomatic patients presenting to specialist breast centres with normal clinical breast examinations. How frequently are these patients undergoing imaging? What is the diagnostic yield? Does yield vary with presenting complaint or age? Do benefits outweigh harms within this population, or a subset? Is this a justifiable use of resources?

**Methods:** This retrospective observational study examines consecutive new attendances at Beaumont Breast Centre over 8 years. All patients with normal clinical examination at presentation were included.

**Results:** 12,426 visits included (98% female) – 10067/12426 (81%) had imaging. Imaging: benign [R1/2] – 9453/10067 (94%), warranting biopsy but likely benign [R3] – 483/10067 (5%), and suspicious [R4/5] – 132/10067 (1%). Biopsy was performed in 713 patients – yielding 554 benign (B1/2), 72 indeterminate (B3/4), and 87 malignant (B5). 96 malignancies were diagnosed in the study population following surgical management, yielding an incidence of 9.5 per 1000 [95% CI: 7.8–11.6] in those imaged. Subgroup analysis for < 45yrs: incidence – 3.9/1000 [95% CI: 2.5–6.3], negative biopsy rate – 94.5%, total cost €2.5 M. High-risk personal/family history was the only presenting complaint associated with increased risk, OR 19.00 [95% CI: 4.16–86.74]. CONCLUSION: Suggest ≥ 45yrs be used as cut-off for imaging given a normal exam, except in those with high-risk history, in line with current asymptomatic screening guidelines.

## O3. Comparison of abbreviated breast MRI with full protocol breast MRI in women with a high familial risk of breast cancer

### **Dr John Lenihan**^1^, Dr Jennifer Kerr^1^, Dr Neasa Ni Mhuircheartaigh^1^, Dr Deirdre Duke^1^, Dr Marie Bambrick^1^, Dr Niamh Hambly^1^

#### ^1^Beaumont Hospital, Dublin 9, Ireland

*Breast Cancer Research* 2024, **26(1)**:O3

**Aim:** Full diagnostic protocol MRI (FDPMRI) is labour intensive from image acquisition and interpretation perspectives. Studies suggest an abbreviated MRI protocol (AbMRI) provides similar accuracy to FDPMRI with significantly lower acquisition and reporting times. We aimed to assess this in a population with a high familial risk of breast cancer.

**Methods:** A retrospective enriched cohort study was performed using a database of women with a high familial risk of breast cancer undergoing surveillance MRI. Patients were assigned to one of two groups of 50 patients, each group containing at least two patients with MRI-detected biopsy-proven malignancy. An abbreviated series consisting of pre- and post-contrast T1 weighted sequences and subtraction reconstructions was reviewed by one of two fellowship-trained breast radiologists. Each radiologist reviewed one group of 50 patients. MRIs designated as RCR category 0, 3, 4, or 5 were deemed positive. A negative study was considered RCR category 1 or 2.

A T2-weighted sequence was subsequently reviewed to determine if this altered management.

**Results:** Results from the two AbMRI groups were pooled. AbMRI and FDPMRI were concordant in 76% of patients (76/100). Recall rate for FDPMRI was 36% (36/100) and 32% (32/100) for AbMRI. All cancers (5/5) were identified as requiring recall on AbMRI. Addition of a T2-weighted sequence to AbMRI altered management in 3% (3/100).

**Conclusion:** AbMRI is reliable in detecting breast cancer in a high familial risk surveillance group. The addition of a T2-weighted sequence rarely alters interpretation.

## O4. Evaluation of Targeted Axillary Dissection in Breast Cancer Patients following Neoadjuvant Chemotherapy: From biopsy to excision

### **Dr Anum Pervez**^1^, Dr Stephan Edey^1^, Dr Ravindran Karthigan^1^, Dr Farah Aldaher^1^, Dr Vasileios Korakis^1^, Dr Rachael Manning^1^, Dr Sultana Hasso^1^, Dr Katerina Ntailiani^1^, Dr Hashim Hamed^1^, Dr Ali Sever^1^

#### ^1^Guy's and St Thomas' NHS Foundation Trust, Great Maze Pond

*Breast Cancer Research* 2024, **26(1)**:O4

**Background:** Targeted axillary dissection (TAD) offers less invasive surgery and more individualised axillary management. It can be an alternative to axillary clearance in node-positive patients who respond to neoadjuvant chemotherapy (NAC). However, identifying appropriate candidates for a TAD procedure is critical. This study is a multi-modality review of axillary imaging with correlation to the surgical outcomes TAD patients. The validity of the pre-treatment marking of the metastatic axillary node is also assessed.

**Methods:** Between 2018 and 2022, breast cancer patients with biopsy-proven axillary disease who received NAC and subsequently underwent a TAD and sentinel lymph node (SLNB) were retrospectively identified.

**Results:** 37 patients met the inclusion criteria. The baseline US identified positive nodes in 92% (34/37) with 100% detection on MRI. On the post-treatment US, 89% (34/37) demonstrated complete imaging response. The pre-treatment clip used for localisation allowed for successful identification of the involved node in 97% of cases (35/36). 49% (18/37) of patients had complete response to NAC with no evidence of nodal involvement on the axillary specimen histopathology. Of the 51% (19/37) who did demonstrate residual disease, a completion axillary clearance was performed with only 3 cases showing disease in the remaining nodes.

**Conclusion:** Approximately half of the node-positive patients treated with NAC can avoid an axillary nodal clearance (ANC) by being offered a TAD procedure instead. As the paradigm shifts towards minimally invasive surgery, the role of the radiologists will become ever more crucial in identifying suitable candidates for this procedure.

## O5. A multicentre review of the direct access mammography programme in Ireland for women with breast pain

### **Dr Caoimhe Geoghegan**^1^, Dr Michelle Horan^1^, Dr Emily Crilly^2^, Dr Aisling Kelly^3^, Dr Rachel Lyons^4^, Dr Laoise Geoghegan^1^, Dr Deirdre Duke^2^, Dr Laura Sweeney^3^, Damian MacCartan^4^, Dr Sylvia O'Keeffe^1^

#### ^1^Radiology Department, St James's Hospital, Ireland; ^2^Radiology Department, Beaumont Hospital, Ireland; ^3^Radiology Department, University Hospital Waterford, Ireland; ^4^Breast Surgery Department, St Vincent's Hospital, Ireland

*Breast Cancer Research* 2024, **26(1)**:O5

Link to published abstract

**Posters**

*Alphabetical by presenting author surname*

## P1. Breast lesions detected at lung screening (Targeted Lung Health Check CTs): impact to breast services and a pathway for management

### **Dr Anuradha Anand**^1^, Dr Lyndsey Brierley^1^, Dr Aninda Saha^1^, Dr Naveed Altaf^1^

#### ^1^North Tees Breast Screening Unit, Stockton, United Kingdom

*Breast Cancer Research* 2024, **26(1)**:P1

**Background:** Targeted Lung Health Check (TLHC) is a national program for detection of early lung cancer, rolled out as a series of pilot programmes, based on lung cancer mortality rates. High risk patients between 55 and 74 years, receive a low dose CT thorax. Inevitably there are significant numbers of incidental breast lesions detected on these scans. In our region, the TLHC catchment area overlaps significantly with the breast screening catchment area.

**Method:** Our unit started receiving referrals from TLHC in December 2022. Recognising the need for a filtering process for these referrals, we set up a pathway where the lead chest physician emails our team with details of patients with incidental breast findings. Our team reviews the patient’s previous screening and symptomatic imaging. If the lesion is longstanding, unchanged or proven benign, we advise no further action. If new or enlarging, we advise assessment in symptomatic clinic.

**Results:** In 7 months, 37 requests for imaging review have been made from 2035 women screened by TLHC.

9 out of 37 were recalled for assessment. Of 9 recalled, 4 were cancer, 4 benign and 1 is awaiting assessment.

All 4 patients diagnosed with cancer had not attended multiple breast screening appointments. Of the 4 benign cases, 2 had no previous mammograms and 2 showed change from previous mammograms.

**Conclusion:** This pathway is effective in preventing unnecessary referral to the symptomatic breast services.

TLHC CTs could be useful in detecting breast cancers in women who do not attend their screening mammograms.

## P2. 5 year retrospective analysis of Vacuum Assisted Excision for B3 breast lesions at University Hospital Southampton, UK

### **Dr Charis Brook**^1^, Dr Ruth Walker^1^, Dr Selina Lam^1^, Dr Brenna Fielding^1^, Dr Monica Banerjee^1^

#### ^1^University Hospital Southampton, Southampton, United Kingdom

*Breast Cancer Research* 2024, **26(1)**:P2

We present our Vacuum Assisted Excisions data performed for B3 breast lesions over 5 years. Management guidelines for B3 lesions introduced in 2016 by the NHS Breast Screening Programme (NHS BSP) addressed concerns of overtreatment, recommending Vacuum Assisted Excision (VAE) in the majority of B3 lesions instead of surgery.

VAE is a well-tolerated procedure that allows adequate sampling to ensure no concurrent malignancy.

**Methods:** All patients who underwent VAE from September 2017 to October 2022 in our breast unit were analysed. Initial histology was compared with histology at VAE, to look for upgrade. Results were compared with published upgrade rates from NHSBSP.

All subsequent recurrence/development of breast malignancy following VAE in the 1–5 years following their procedure was reviewed.

**Results:** 244 VAEs were performed. Overall upgrade rate of B3 lesions was 10.6%. The most frequently upgraded histology was AIDEP (28%).

Most patients (92%) undergoing VAE were of screening age population (47–73 yr old). 19 patients < 50yrs underwent VAE following symptomatic presentation.

3 patients (1.2%) who were discharged following VAE with non-upgraded histology presented with malignancy within 5 years of VAE. All these were under 50 yrs at the time of VAE. We have since modified our practice and do not perform VAE for B3 lesions in patients under 50yrs old.

**Conclusion:** VAE allows adequate sampling of B3 lesions and is both less invasive than surgery, and provides cost saving to a stretched NHS. VAE can provide an effective, definitive, and well-tolerated treatment of B3 breast lesions.

## P3. Analysis of the current mammographic surveillance programme post primary breast cancer in a symptomatic breast centre

### **Emily Crilly**^1^, Dr Matthew Common^1^, Dr Jack Horan^1^, Aisling Hegarty^2^, Professor Jan Sorenson^2^, Eithne Downey^1^, Dr Deirdre Duke^1^

#### ^1^Beaumont Hospital Radiology, Dublin, Ireland; ^2^Royal College of Surgeons of Ireland, Dublin, Ireland

*Breast Cancer Research* 2024, **26(1)**:P3

**Background:** Mammography is the surveillance test of choice post primary breast malignancy. Current Irish guidelines are for annual mammography until the age of 70 or for 5 years post-surgery, whichever is later. This generates significant workload and has little evidence to support it.

**Aim:** To analyse post breast cancer mammographic surveillance in a single symptomatic breast centre, with reference to potential areas of improvement.

**Methods:** All patients diagnosed with breast cancer between Jan 2010–Dec 2014 were identified. Histological subtypes and receptor status was recorded. Number of routine surveillance mammograms, recalls and subsequent investigations were recorded and interpreted using descriptive statistics and logistic regression models.

**Results:** 1,491 patients were diagnosed with breast cancer over 5 years. 69 were excluded and 1,083 removed from surveillance. Of those removed, 35% developed metastases, 21% reached 70 + , 13% were managed medically, 13% had bilateral mastectomies and 18% were medically unwell.

339 patients were still undergoing mammographic surveillance in 2022, generating 3,109 single-read mammograms since diagnosis. There were 321 recalls for further imaging and 46 biopsies. Recall rate within the first 5 years is significantly higher compared to years thereafter; odds ratio 0.5 (p value = 0.00, CI 0.39–0.64). There were 12 malignant biopsies within 3,109 routine mammograms (0.38%).

If the surveillance interval reduced to biennial after 5 years, workload would be reduced by 934 mammograms and identification of 4 malignancies would have been delayed by 1 year.

**Conclusion:** A review of current guidelines should be considered accounting for patient age, histological subtype and family history.

## P4. A Review of Patient Outcomes at a Low Risk Breast Clinic

### **Aisling Daly**^1^, Dr Elaine Davis^1^

#### ^1^Belfast City Hospital, Belfast, Northern Ireland, United Kingdom

*Breast Cancer Research* 2024, **26(1)**:P4

**Aim:** We describe the outcomes in all non-urgent referrals to our Regional Centre triaged as "routine" and aged under 30 years of age.

**Background:** Breast cancer is rare in young women. The incidence of breast cancer in women younger than 30 has been recorded as 0.43% and in Western countries, women under the age of 30 account for 0.65% of all breast cancers.

This low incidence rate provides the rationale behind avoiding unnecessary radiation and biopsy in women under 30 years of age presenting with low risk symptoms.

**Method:**

1. Retrospective analysis of all referred patients to our institution under the age of 30 over the previous 7 years. Red flag referrals were excluded.

2. Non urgent referrals were based on the NICAN (Northern Ireland Cancer Network) guidelines where "Non urgent" is considered in patients under 30 presenting with an unexplained lump with/without pain.

3. Clinics were staffed by one Staff Grade in Breast surgery and two breast sonographers.

4. Main presenting complaint was a mobile breast lump (± pain).

**Results:** We found 3 cases of breast cancer out of a total of 1300 patients under the age of 30 who had been referred to the low risk breast clinic at our institution over 7 years.

**Conclusion:**

- Patients under the age of 30 can be safely managed in low risk breast clinics.
- Low risk clinics allow timely review and in the vast majority of cases, patients are reassured and discharged.

## P5. MRI Breast Cancer Surveillance-service evaluation

### **Dr Georgina Devenish**^1^, Francesca Moakes^1^

#### ^1^Royal United Hospital Bath, Bath, United Kingdom

*Breast Cancer Research* 2024, **26(1)**:P5

**Background:** MRI is highly sensitive in the detection of breast cancer. Although there are no established national guidelines for MRI in breast cancer surveillance (BCS), it may be considered in young women, dense breasts and mammographically occult disease. Locally these indications are incorporated and MRI is used alongside mammography for a minimum of 3 years.

**Aims:** To evaluate our indications for BCS MRI and establish a standardised local protocol for indications and duration of MRI surveillance. Also to analyse our cancer detection using MRI for BCS.

**Method:** Retrospective review of patients who underwent MRI for BCS over a 10 year period. Clinical indications, histopathology and year of recurrence/new disease were recorded.

**Results:** 44 patients identified over the 10 year period. Median age 48.4, median duration of surveillance 2.7 years. Most common indication was occult disease.

8/44 (18%) were recalled from MRI surveillance for assessment. 3 did not need further intervention. 5 of these underwent biopsy. 2/5 were MRI detected recurrence/new disease > 3 years MRI BCS. 3/5 were benign/B3.

A further 4 recurrences were symptomatic presentations at years 3, 5, 8, 14 respectively.

Histopathology of 6 recurrences revealed 2 ILC and 4 IDC.

**Conclusion:** The indication of mammographically occult disease revealed the highest percentage of recurrent/new disease. 83.3% of the cancers detected were > 3yrs post diagnosis, therefore potential benefit of MRI BCS extended to the same duration as mammography criteria.

Tumour type, age and family history may influence the eligibility of BCS MRI, with further research needed to standardise guidelines.

## P6. Comparative analysis of salient imaging features of breast lesions on Mammograms, MRI, and Ultrasound in High-risk breast cancer women for early detection of cancer

### Dr Neelam Soni^1^, **Dr Asha Eleti**^1^

#### ^1^Department of Radiology, Southend University Hospital, Mid and South Essex NHS trust, Southend-on-sea, United Kingdom

*Breast Cancer Research* 2024, **26(1)**:P6

**Aim:** Compare and accurately characterize lesions on mammograms, MRI and ultrasound in high-risk breast cancer women for early detection of cancer.

**Method:** Retrospective data obtained from high-risk breast cancer screening women (249) from last 5 years. Total 40 cases, with findings on screening mammograms/MRI, were recalled for triple assessment and included in study. Women with recommendation of routine annual recalls were excluded. All modality imaging findings were graded on standardized 5-point system and correlated with histopathology.

**Results:** Mean age was 45.1 with 60% BRCA 1, 32.5% BRCA 2, 5% PALB2 and 2.5% TP53. Total 37.5% recalled from screening MRI and 22.5% from mammograms. Histopathology revealed, 25% of indeterminate lesions on mammograms (M3), 55% on ultrasound (U3) and 30% on MRI (MRI3) turned out to be malignant.

Common findings on mammograms were mass (27.5%), microcalcifications (17.5%) and distortion (10%). On ultrasound, malignant lesions were frequently well-circumscribed (52.5%) rather than typical spiculated (15%) or microlobulated (12.5%). Additionally, only 20% demonstrated anti-parallel orientation. On MRI, majority were well circumscribed (40%) and 12.5% non-mass enhancement.

Total 40% of indeterminate and suspicious lesions on MRI were normal on mammograms, due to extremely dense breast parenchyma. Furthermore, 2 women with marked background parenchymal enhancement and no specific lesions on MRI were subsequently diagnosed cancer on risk-reducing mastectomies.

**Conclusion:** Malignant lesions in high-risk women often appeared indeterminate on all modalities, and typical malignant features were infrequent. Biopsy at initial instance and dedicated screening MRI/mammograms are crucial for early detection and management of cancer in this group.

## P7. CT staging in breast cancer- are we over staging?

### **Dr Lucinda Frank**^1^, Dr Rebecca Geach^1^

#### ^1^North Bristol NHS Trust, Bristol, United Kingdom

*Breast Cancer Research* 2024, **26(1)**:P7

**Introduction:** Following an initial audit, our local guidelines for CT staging in breast cancer were updated in November 2021. This re-audit aimed to evaluate compliance with the updated local guidelines and compare compliance with the initial audit.

**Method:** A retrospective audit was conducted of 100 staging CT chest/abdomen/pelvis between 16th September 2022 and 31st December 2022. This included reviewing the indication for the staging CT and the percentage detection rate of metastatic disease.

**Results:** Fifteen cases had a previous history of breast cancer and 2 scans were for follow-up, these were therefore excluded.

66/83 cases clearly met the updated guidelines. Four CT scans were requested pre-operatively for several abnormal axillary lymph nodes however the exact number of nodes was not specified. One case, an aggressive papillary lesion progressed rapidly, this didn’t meet the guidelines initially however the patient required NACT. Twelve cases did not meet the guidelines, none of these had metastatic disease.

Eleven patients had bone/liver/lung metastases. Further imaging was recommended in 50% cases.

**Conclusion:** 80% staging CTs met the locally agreed guidelines. This is unchanged from the initial audit in 2020 and demonstrates the challenge in achieving a sustained change in practice.

14% cases didn’t meet the guidelines and none of these patients had metastases. From a radiology perspective clearly documenting the number of abnormal axillary lymph nodes would help ensure the correct patients have staging. 50% of cases required further imaging which significantly increases the radiology workload. Indeterminate results also lead to continued patient anxiety.

## P8. Breast MRI in the absence of MRI-guided biopsy: A retrospective review with three years follow-up data

### **Dr Soheila Hajialiasgar**^2^, Dr Sarah Savaridas^1^

#### ^1^University of Dundee, United Kingdom; ^2^NHS Tayside, Dundee, United Kingdom

*Breast Cancer Research* 2024, **26(1)**:P8

**Background:** Utilisation of breast MRI is ever-increasing against a backdrop of workforce shortages. MRI has high sensitivity, but lower specificity may result in unnecessary recalls and benign biopsies. This is further hampered by extremely limited access to MRI-guided biopsy.

**Methods:** Clinical reporting systems were used to identify all breast MRIs performed between 01/01/2019–31/12/2019. Indication for MRI and MRI lesion features were recorded, recall rates and biopsy rates were calculated. Pathology was reviewed. Cases were followed-up for a minimum of three years.

**Results:** 283 MRIs (216 women) were included. Indications were; high-risk screening(40), neoadjuvant chemotherapy monitoring(65), locoregional staging(89) and other(22). Twenty-seven MRIs (9.5%) were recalled. Twenty-six biopsies were performed, biopsy results were B1-2 (4), B3 (4), B5 (19). One B3 case was upgraded to ILC at surgery. Cancer detection rate in the screening group was 7.5% (3 of 40). Two cancers were identified in the follow-up period: one in a high-risk screener, one presumed previously occult on all imaging with proven nodal disease and contralateral cancer. No subsequent cancers were found in the recalled cases with normal/benign second-look imaging and/or biopsy.

**Discussion:** When MRI is conducted in a carefully selected population, recall rate and benign biopsy rate is relatively low. Follow-up data is reassuring, with no evidence that the lack of access to MRI-guided biopsy resulted in missed cancers.

## P9. Breast arterial calcifications on mammography; updates on it’s significance, qualitative report descriptors and management

### **Dr Aine Heaney**^1^, Dr Roisin Heaney^1^

#### ^1^St James's Hospital, Dublin, Ireland

*Breast Cancer Research* 2024, **26(1)**:P9

Background: Cardiovascular disease (CVD) is the leading cause of premature death for women in the United Kingdom with twice as many women dying from coronary heart disease (CHD) than breast cancer. It is estimated that healthcare costs relating to heart and circulatory diseases cost the UK economy £9 billion each year. Breast arterial calcification (BAC) is an incidental finding on mammography that is strongly correlated with coronary artery calcification (CAC) on computed tomography (CT) and incident CVD events including CVD death and ischaemic stroke. By identifying those with BAC, we can identify women who are at higher cardiovascular risk and potentially intervene at an earlier stage to prevent adverse cardiovascular events and outcomes and reduce the economic burden. Currently there are no standardised guidelines for the reporting of BAC on mammography in the UK and Ireland.

Learning Objectives

1. To review the most recent literature on the association between breast arterial calcification and CVD outcomes as well as the understanding and reporting habits of breast radiologists in Europe, Canada and the USA.
2. To highlight and describe a quick and easy qualitative BAC grading system that has been previously published in the literature, with suggested wording for mammogram reports, and which has recently been endorsed by the Canadian Society of Breast Imaging (CSBI).
3. To review the current management recommendations for BAC on mammography as described by cardiologists in the literature.

## P10. Stereotactic biopsy of microcalcification using standard 14- and 12-G core biopsy versus vacuum-assisted biopsy during a global needle shortage

### **Dr Chao Jin Ho**^1^, Dr Alice Leaver^2^, Dr Alan Redman^2^, Dr Simon Lowes^2^

#### ^1^Gateshead Health NHS Foundation Trust, Gateshead, United Kingdom; ^2^Breast Screening and Assessment Unit, Gateshead Health NHS Foundation Trust, Gateshead, United Kingdom

*Breast Cancer Research* 2024, **26(1)**:P10

In 2021 there was a global shortage of VAB (vacuum-assisted biopsy) needles, meaning our unit had to start using standard core biopsy (CB) needles (14- and 12-gauge) for some of our stereotactic biopsies. This study assesses the success of this technique by using B1 rate and B3 upgrades of VAB versus CB.

Data were collected retrospectively for all stereotactic biopsies of microcalcification during 2021 using MDM records and pathology reports.

There were 300 stereotactic biopsies for microcalcification in 283 women. 193 (64.3%) were VABs and 107 (35.7%) were CB. Although both techniques yielded a similar proportion of B2, B3, B4, B5a, and B5b results (59.1, 13.0, 0.5, 21.8, 3.1% versus 57.0, 9.3, 0.9, 19.6, and 2.8% for VAB and CB, respectively), there were fourfold more B1 results for CB compared to VAB (10.3% versus 2.6%).

Of the eleven B1 CB results, 10 underwent VAB, yielding seven B2s and three B3 with atypia, one of which was malignant at VAE.

Of ten initial B3 CB results, 6 had atypia initially, of which 4 were upgraded at VAE to malignancy. Of the 4 with no atypia initially, 2 were upgraded to atypia at VAE.

Of twenty-five initial B3 VAB results, 20 had atypia, one of which was upgraded to malignancy at VAE. Of the 5 no-atypias, 1 was upgraded to atypia at VAE.

In conclusion, CB yielded fourfold more B1 results than VAB, resulting in additional biopsies. To some extent this may represent a learning curve to adopting a new biopsy technique.

## P11. Single reader cancers – where are they found?

### Dr Marianne Kimura^1^, **Dr Luci Hobson**^1^, Michelle Taylor^1^, Dr John Gifford^1^, Dr Nicholas Ridley^1^, Dr Karen Litton^1^

#### ^1^Great Western Hospitals NHS Foundation Trust, Swindon, United Kingdom

*Breast Cancer Research* 2024, **26(1)**:P11

The NHS breast screening programme has mandatory double reporting of mammograms.

Some cancers will only be detected by a single reader. These films are by nature those that are tricky to interpret with less obvious features to perceive.

This is a review of the mammographic features, site, size, and tumour types of these cancers.

In this study we reviewed all the single reader called cancers over a period of 5 years. (between 04/2017-04/2022).

No differentiation was made between job role of the reporter or the order of film reading.

A total of 132 patients had a cancer diagnosed by a single reader.

25% of these were non-invasive and 75% were invasive.

The main feature (78%) of non-invasive disease was calcification and over half ( 56%) of the non-invasive disease was high grade.

The main features of the invasive disease were ill defined masses or distortions. 28% of the invasive disease was grade 3. The mean size of the radiographic features detected was less than 13 mm.

The locations of the radiographic abnormalities will be mapped onto a large diagram on the poster using symbols for features.

O—well defined mass.

X—ill defined mass,

#—asymmetry.

*—distortion and.

▲—calcification.

48/132 of the features were found in one of the typical review areas for mammography.

The aim of the poster is to highlight these review areas and features of these difficult to perceive malignancies and to highlight these areas to film readers.

## P12. Architectural distortion on digital tomosynthesis mammograms in symptomatic Breast clinics: Correlation with pathological outcomes

### **Riya Kale**^1^

#### ^1^Cardiff University, Cardiff, United Kingdom

*Breast Cancer Research* 2024, **26(1)**:P12

**Aim:** In our centre, standard two-dimensional mammograms were replaced with Digital Breast Tomosynthesis (DBT) and synthesized 2D views (C view) for symptomatic ‘one-stop’ clinics from April 2020. The aim of this study was to evaluate the positive predictive value for malignancy in DBT detected Architectural Distortion (AD).

**Methods:** All mammogram reports with the word ‘distortion’ between April 2020 to October 2022 were assessed. There was a total of 458 mammograms with the word ‘distortion’. After excluding mammograms with no distortion (n = 128), post-surgical distortion (n = 173), distortion with mass (n = 33) and unchanged distortion (n = 14), there were 111 patients with pure distortion, which were included in this study. A flow chart depicting patients’ journey and the management pathway was created. Correlation with histopathology was obtained. All patients were followed for at least two years.

**Results:** Forty-two out of 111 patients (37.84%) with AD had a normal ultrasound and were discharged. Fifty-five (49.5%) had US correlation corresponding to the distortion, leading to US-guided-biopsy, and 13 (23.6%) had Tomosynthesis-guided-biopsy. One had the diagnosis made by skin punch biopsy. The positive predictive value (PPV) for malignancy was 42.34%. Malignancy diagnoses were higher with US-guided biopsies than with Tomosynthesis-guided biopsies, 78.1% and 30%, respectively. A total B3 rate of 9.9% (38% and 10.9% with tomosynthesis guided and US guided biopsies respectively.

**Conclusion:** A total malignancy rate of 42.34%, DBT detected architectural distortion has a high enough PPV for malignancy to justify tissue sampling. Chances of malignancy are higher when a US correlate corresponding to AD is present.

## P13. Cancer incidence in a 7 year follow up of a B3 cohort—Can we use breast screening for over 50's?

### Dr Yuxuan Zhang^1,2^, **Dr Gerald Lip**^1,2^

#### ^1^NHS Grampian, Aberdeen, United Kingdom; ^2^University of Aberdeen, Aberdeen, United Kingdom

*Breast Cancer Research* 2024, **26(1)**:P13

**Introduction:** Lesions of uncertain malignant potential in the breast (B3) are currently followed up via enhanced surveillance with yearly mammograms for five years after initial large volume excision.

**Methods:** A retrospective review was carried out in June 2023 on all breast screening patients on enhanced surveillance following B3 histology that were managed with large volume excisions in a Scottish Breast Screening unit from 2016 to 2018.

All cases were followed up as per London Breast Screening QA guidance initially and then the NHS Breast Screening Guidance after as guidelines evolved. Patients both 5 years were confirmed as being followed up via patient records and breast screening records.

**Results:** 9 cases were excluded due to subsequent upgraded histology. 3 patients moved out of catchment area during follow-up period. 2.9% (3/104) of patients developed breast malignancies at the time of follow up. 1 patient developed invasive ductal carcinoma in year 3, whilst 2 patients developed invasive ductal carcinoma and lobular carcinoma respectively in year 4.

**Conclusion:** The malignancy rate amongst our local screening post large volume excisions B3 patient cohort is low. Current practice of intensive yearly follow up over a 5-year period may not be the most appropriate way of utilising limited breast radiology resources. Wider discussion on follow up timing of these slow-developing lesions via the breast screening programme, or staggered over a longer time frame should be considered. Our data supports follow up mammography could be done at the regular screening interval. Anticipated results from the Sloane cohort study should provide further clarification on this matter in due course.

## P14. Change of arbitration strategy in an NHS BSP unit to include double reader recalled mammograms following change of target recall rate: effect on recall and cancer detection rates

### Richard Morrell^1^, **Dr Simon Lowes**^1^, Allison Wise^1^, Dr Alan Redman^1^, Dr Gurpreet Hamilton^1^, Dr Sally Athey^1^, Dr Alice Leaver^1^

#### ^1^Gateshead Health NHS Foundation Trust, Gateshead, United Kingdom

*Breast Cancer Research* 2024, **26(1)**:P14

**Background:** NHS BSP target recall rates decreased in April 2021. We therefore added third reader arbitration of double reader recalled screening mammograms to our long-standing practice of arbitrating all single reader recalls.

This study aims to explore the effect of these changes upon recall rate and cancer detection rate (CDR).

**Method:** Recall rate and CDR data for all NHS BSP patients screened in our unit were determined from NBSS crystal reports over three time periods (01/07/2016–31/06/2017, 1/07/2018–31/06/2019 and 01/07/2021–31/06/2022) to improve confidence that any rate change was due to the changes implemented. Descriptive statistics were performed.

**Results:** Our NHS BSP unit serves a population of 120,000 women. This data includes results from nineteen readers; seven readers contributed to all time periods examined.

Overall recall rates and CDR were 6.2% prevalent, 2.9% incident with a 1.27% average CDR between 01/07/2016–31/06/2017, 7.1% prevalent, 3% incident with a 1.32% average CDR between 1/07/2018–31/06/2019 and 4.6% prevalent, 2.1% incident with a 1.65% average CDR between 01/07/2021–31/06/2022.

**Conclusion:** Results suggest a decrease in overall unit recall rates following the change in NHS BSP target recall rates and our extension of arbitration to include double reader recalls. Cancer detection rate did not fall. Implementation of both changes simultaneously has meant that the relative contribution of each change cannot be determined.

## P15. The last decade of breast MRI in a regional breast unit: change in demand and clinical indications

### Dr Joanne Swithenbank^1^, Dr Preet Hamilton^1^, Richard Morrell^1^, Dr Alan Redman^1^, Dr Alice Leaver^1^, **Dr Simon Lowes**^1^

#### ^1^Gateshead Health NHS Foundation Trust, Gateshead, United Kingdom

*Breast Cancer Research* 2024, **26(1)**:P15

**Introduction:** There is increasing pressure on all breast imaging services, including MRI. This study evaluates the number of breast MRI examinations performed over an 11-year period spanning January 2012 to December 2022, and any trends in the clinical indications.

**Methods:** Contemporaneous records of breast MRI examinations were analysed over the study period. Descriptive statistics were performed including analysis of primary indications for scanning.

**Results:** Between 2012–2019 there was a year-on-year increase in the number of MRI scans performed, from 60 in 2012 to 288 in 2019. In 2020, the number dipped to 186, likely secondary to the COVID-19 pandemic, but this has gradually risen again to 278 scans in 2022. Between 2012–2016, the principal indication for MRI was lobular carcinoma, accounting for an average of 38.3% of examinations over that period (range 31.7–41.7%), with neoadjuvant chemotherapy (NAC) accounting for 5.1% of scans over the same timespan (range 1.7–9.7%). In 2017, the proportion of examinations performed for assessment of NAC increased sharply to 28.2% and it continues to show a year-on-year increase. Spanning 2017–2022, NAC assessment now accounts for an average 35.2% of all scans (range 28.2–38.8%). The second commonest indication for MRI is now lobular carcinoma (20.1% in 2022) then surveillance (16.2%) and occult disease (12.9%).

**Conclusion:** The 4.8-fold increase in MRIs between 2012–2019 largely reflects an increased use of NAC, but workload for other indications has also increased. This trend was interrupted during the COVID-19 pandemic, but seems to have recovered and reached a plateau for the time being.

## P16. Impact of breast density on preoperative breast MRI on evaluation of invasive lobular carcinoma

### **Dr Lauragh Mc Carthy**^1^, Dr Elisabetta Giannotti^1^, Dr Yan Chen^2^, Dr Nuala Healy^1^, Dr Fleur Kilburn-Toppin^1^, Dr Elena Provenzano^1^, Professor Fiona Gilbert^1^

#### ^1^Cambridge University Hospitals, United Kingdom; ^2^University of Nottingham, United Kingdom

*Breast Cancer Research* 2024, **26(1)**:P16

**Purpose:** Guidelines recommend pre-operative MRI in invasive lobular carcinoma (ILC) irrespective of breast density.

The aim of the study was to assess the performance of MRI in preoperative staging and determine if breast density has an impact.

**Methodology:** Retrospective study of ILC from 01/2015 to 12/2017 who had a preoperative MRI. Mixed invasive lobular and invasive non special type carcinoma were excluded. From mammograms patients were divided into low breast (A + B) and high breast density (C + D).

Index lesion size and multifocality were recorded for mammography and MRI.

**Results:** 73 cancers had paired histology and MRI lesion size measurements. Histology (M = 32.4 mm, SD = 30.7 mm) and MRI (M = 30.7 mm, SD = 23.3 mm) lesion size measurements did not differ significantly; the small effect size indicated statistical equivalence (Cohen’s d = 0.085, 90%CI −0.092 to 0.261). Paired histology and MRI lesion sizes did not differ significantly for cases of “A or B” or cases of “C or D” density.

Histology and MRI lesion size measurements exhibited strong correlations across all cases with paired measurements, and when cases of “A or B” and cases of “C or D” density were considered separately (all r values > 0.7). The strength of correlation did not differ significantly between cases of “A or B” and cases of “C or D” density.

MRI sensitivity for multifocality detection was 39.06% with 77.8% specificity, with no significant differences in sensitivity or specificity between cases of “A or B” and “C or D” density.

**Conclusion:** MRI is accurate for preoperative assessment of ILC and should be performed in all breast densities.

## P17. The Management of Male patients in the Symptomatic Breast Clinic-the effectiveness of imaging in the diagnosis of breast cancer

### **Elaine Mcguckin**^1^

#### ^1^Southern Health and Social Care Trust, Craigavon Area Hospital, United Kingdom

*Breast Cancer Research* 2024, **26(1)**:P17

**Purpose:** To review the number of males attending the Breast Unit from January 2016–December 2022, and evaluate their imaging and clinical outcomes to assess if there is a clear pathway in place to effectively manage male patients.

**Method:** A retrospective study was made of the clinical and imaging records of all symptomatic male patients presenting to Breast Unit for imaging over a 7 year period.

**Results:** A total of 1190 male patients aged 22–96 years were referred. After clinical examination, 1101 underwent imaging.

In total 289 (26%) had mammography, 845 (77%) had ultrasound and 211 (19%) had both mammography & ultrasound. Interventional procedures were performed on 71 (6%) including a total of 59 core biopsies and 25 FNAs. 200 (18%) under 40 years were imaged (196 ultrasound, 4 mammogram only and 2 males both ultrasound & mammogram).

Only 11 (0.9%) were diagnosed with primary breast carcinoma, 746 (67%) were diagnosed with gynaecomastia with 130 (65%) of those being under 40 years. 50% of total males diagnosed had clinical symptoms attributed to medication, recreational drugs, dietary supplements & lifestyle choices.

**Conclusions:** The overwhelming majority of male breast problems identified are benign, the most common being gynaecomastia and imaging is not usually recommended. However within the Unit, male referrals create excessive imaging workload especially among under 40 males and with pathways that are not consistent.

It may prove beneficial to develop a MDT Approach Management Strategy incorporating an age related protocol, to streamline the symptomatic pathway and reduce unnecessary imaging.

## P18. Avoiding biopsy for presumed fibroadenomas with benign ultrasound greyscale and shear-wave elastography features in women aged 25–39 years: Comparison between two ultrasound systems and review of follow-up data

### **Dr Kirsty McNeil**^1,2^, Dr Sarah Savaridas^1,2^

#### ^1^NHS Tayside, Dundee, United Kingdom; ^2^University of Dundee, Dundee, United Kingdom

*Breast Cancer Research* 2024, **26(1)**:P18

**Background:** Based on previous research using an Aixplorer ultrasound system, biopsy is not required for presumed fibroadenomas with benign greyscale ultrasound and shear-wave elastography (SWE) findings for women aged 25–39 years. This service evaluation compares findings between the Aixplorer and Samsung ultrasound systems, and reviews follow-up data.

**Methods:** Patients aged 25–39 years who attended the breast clinic between 03/06/21–12/07/23, with presumed fibroadenoma with benign ultrasound and SWE features were included. Age and average SWE value (across four images) were recorded, grey-scale images were reviewed for benignity. The independent T-test was used to compare patient age and average lesion SWE value between those scanned on the Aixplorer system compared with the Samsung system. Databases were reviewed for patients with at least six months follow-up.

**Results:**111 patients were included (Aixplorer cohort: 30 patients, Samsung cohort: 81 patients). Average age was 32.0 years, no significant difference between cohorts (p = 0.4). Average SWE values were significantly higher in the Samsung cohort compared to Aixplorer cohort; 33.19kPA vs 23.27kPA respectively, p < 0.01. 36 patients had six months – 2 years follow-up with no subsequent malignant diagnosis, two further lesions with benign greyscale and SWE features were biopsied and proven to be fibroadenomas.

**Conclusion:** No cancers were detected on follow-up. Average SWE values were significantly higher in the Samsung cohort. This supports that the existing guidance can be safely applied to the new system, however, it suggests that a higher cut-off value may be appropriate to limit unnecessary benign biopsies. Further validation work on the new system is recommended.

## P19. Arbitration of prevalent round recalls in the West Devon & East Cornwall Screening Programme—a re-audit

### **Dr Olubukola Omidiji**^1^, Dr Erika Toth^1^, Dr Sarah Doyle^1^, Dr Karen Paisley^1^, Jen Taper^1^

#### ^1^University Hospitals Plymouth, Plymouth, United Kingdom

*Breast Cancer Research* 2024, **26(1)**:P19

**Background:** This re-audit was performed to assess effectiveness of the action plan formulated following baseline audit of prevalent recall rate (pRCR) in 2016/17, which showed a high pRCR of 12.43%, above the then NHSBSP Acceptable Standard of < 10%. In May 2017, unit policy changed to incorporate a consensus arbitration meeting for all prevalent recalls (unless classified M5 by both readers) to try and decrease pRCR. This audit aims to determine if pRCR is now within the NHSBSP Achievable Standard of < 7%, and to establish if there has been any accompanying change in cancer detection rate (CDR).

**Method:** A list of all prevalent screens from 1 April 2021 to 31 March 2022 was provided by the Screening Programme Manager. Assessment cases from this list were reviewed using NBSS and PACS by one film reader. The number of reads, reason for recall, imaging and clinical scores, biopsy score and histology outcome were documented and analysed using Excel 2021.

**Results:** 2315 prevalent screens were performed, of which 194 (8.4%) were recalled for assessment. This is lower than before introduction of a consensus arbitration meeting, but still above the Achievable Standard. Arbitration occurred in 61.8% cf 46.2% in 2017/18. CDR was 13.8/1000, higher than before policy change (6.2/1000 in 2016/17 and 12.7/1000.

in 2018/19). 17.9% of recalled ill-defined masses were malignant, 21.7% of distortions and 23.5% of calcifications.

**Conclusion:** Consensus arbitration, by a group or at least a 3rd reader, lowered recall rate in the prevalent population, without a fall in cancer detection rate.

## P20. 10-year retrospective review of Male Breast Cancer in a single Breast Cancer Centre

### Dr Lara Toerien^1^, **Dr George Petrov**^1^, Dr Sophie Murphy^1^, Ms Laurentia Berzan^1^, Dr Niamh O'Mahony^1^, Dr Angela O'Brien^1^

#### ^1^Mater Misericordiae University Hospital, Dublin, Ireland

*Breast Cancer Research* 2024, **26(1)**:P20

**Introduction:** Male Breast cancer (MBC) is uncommon, with 1.2 cases per 100,000, however incidence has steadily increased in past decades. Improved understanding of patient characteristics, risk factors, and pathology, and how those differ from female patients, is paramount for early detection and improved treatment.

**Aim:** To determine epidemiological characteristics, imaging findings and histological properties of MBC in our institution, and compare with published literature.

**Method:** Retrospective review of electronic records and imaging of all male patients diagnosed with breast malignancy at our centre from 2012–2022.

**Results:** 4179 patients in total were diagnosed with breast malignancy from 2012–2022. 1% were male. Of those, only 51% were diagnosed with a primary breast malignancy, and 49% with secondary breast metastasis, with lymphoma and melanoma the most common primaries. This contrasts with an 85% primary breast malignancy rate within our female population. Commonest presenting symptoms of MBC were palpable central lumps (54%) and nipple symptoms (42%). Ages ranged from 44–92, with a mean age of 68. 38% had a positive family history, of which 2 were BRCA positive. Smoking and alcohol consumption were reported in 25% and 33% respectively. 33% were node positive. Invasive ductal carcinoma was diagnosed in 83%, with 88% oestrogen/progesterone receptor positive and 92% HER-2 negative, comparable with female receptor statuses described in literature.

**Conclusion:** MBC is rare. The ratio of malignancies that are secondary metastases is higher in the male than female population. Incidence, imaging characteristics and tumour biology of MBC in our institution are similar to previously published studies.

## P21. Measurement of LOCalizer Tag depth for breast cancer surgery

### Dr Oscar Redman^1^, Dr Simon Lowes^2^, **Dr Alan Redman**^2^

#### ^1^Northumbria NHS Foundation Trust, Newcastle Upon Tyne, United Kingdom; ^2^Gateshead NHS Health Foundation Trust, Gateshead, United Kingdom

*Breast Cancer Research* 2024, **26(1)**:P21

**Purpose:** LOCalizer Radiofrequency Identification Tags localise impalpable breast lesions. Radiologists complete a form regarding Tag location for surgeons. The LOCalizer system is unusual because a handheld Reader measures Tag depth. Radiologists document Skin to Tag depth measured by the Reader. Changing to a localisation system without a Reader would necessitate change to ultrasound depth measurements. Radiology team are uncertain 1. whether surgeons value the recorded depth measurement, and 2. whether ultrasound measurements would be significantly different to Reader measurements for the same Tag location. This study aims to answer both questions.

**Method:** 1. Survey of local surgeons: are radiology-recorded Tag depths ‘not at all’, ‘somewhat’ or ‘very’ useful?

2. Retrospective study comparing documented US and Reader skin to Tag measurements. All Tags were inserted between October 2019–September 2022. Measurements analysed for each breast region.

**Results:** 1. Majority of surgeons find radiological documentation of depth either ‘somewhat’ or ‘very’ useful.

2. 156 Tags with US and Reader measurements were included in the retrospective review. Locations: 12 axillae, 10 lower outer quadrants, 16 lower inner quadrants, 74 upper outer quadrants, 37 upper inner quadrants and 7 subareolar regions. Mean measurement for Ultrasound (16.4 mm) was 6.5 mm less than for the Reader (22.9 mm). Similar trend across all regions.

**Conclusion:** Radiology should continue to document device depth pre-surgery.

If we change to a localisation system which does not measure depth, surgeons should know that ultrasound measurements will be approximately 6.5 mm less than the Reader would have given for the same device depth.

## P22. Evaluation of over 71 year old self-referrals in a UK breast screening programme; what is the impact on workload?

### **Dr Wesam Rjoop**^1^, Dr Karen Paisley^1^, Frances Kinsman^1^

#### ^1^University Hospitals Plymouth NHS Trust, Plymouth, United Kingdom

*Breast Cancer Research* 2024, **26(1)**:P22

**Aim:** To review clients ≥ 71-years who self-referred for screening between April 2019 and March 2022, and to evaluate key performance indicators relative to the invited population aged 50–70 years, and impact on workload.

**Methods:** 3 years NBSS outcome data was provided by the Programme Manager and analysed by age cohort. Clients invited through the Age-X trial were excluded. Imaging was reviewed for all self-referral cancers.

**Results:** 2,980 women ≥ 71-years self-referred for screening during the 3 yr study period; this compared to 54,123 screens in invited 50–70-year-olds. While recall rate for self-referrals (5.5%) was similar to invited (4.3%), cancer detection (CDR) was higher in self-referrals (21.14 vs 9.61 per 1000). Consequently, positive predictive value (PPV) was greater in self-referrals (38.4% vs 22.1%).

Most self-referrals had been screened < 5-years previously (82.4%); although, interestingly, 17.5% were previous non-attenders.

Regarding workload, clients ≥ 71-years represented a 7.8% increase in numbers screened, a 9.3% increase in recalls, and a 10.9% increase in biopsies.

63 cancers were detected in self-referrals. The main mammographic feature was a mass (80%). Histology was invasive in 81%. 20 cancers were in clients ≥ 80-years (2 in situ only). 79% underwent WLE. 30 cancers were palpable (47.6%); 15 were P4-5 (23.8%).

**Discussion:** While self-referrals accounted for only a small proportion of those screened (5.2%), CDR was much greater in self-referrals cf invited.

A high proportion of cancers in self-referrals were palpable, suggesting this might have influenced decision to self-refer.

33% self-referral cancers were in ≥ 80 years (oldest 87 yo) raising issue of over-diagnosis.

## P23. Cancers detected from Consensus Arbitration

### **Christine Robinson**^1^, Dr Norlinda Johnston

#### ^1^Southern Health and Social Care Trust, Portadown, United Kingdom

*Breast Cancer Research* 2024, **26(1)**:P23

**Purpose:** Double reading screening mammograms without arbitration does increase the recall rate but can increase cancer detection rate by 6%–15% when compared with single reader reporting. To reduce unnecessary recalls, a process of consensus or arbitration can be used for discordant results. Outcomes of all women discussed at consensus meetings over a two-year period were reviewed identifying cancer detection rate, breast cancer types and imaging characteristics.

**Methods and Materials:** A retrospective review of all arbitrated screening mammograms in a single breast screening unit from January 2021 to December 2022. All arbitrated mammogram results were recorded as return to routine screening or recall to assessment. Assessment outcomes were reviewed for cancer types and cancer mammographic features. Interval cancer rates were regularly monitored to ensure standards were met.

**Results:** A total of 815 women with discordant mammographic findings were discussed at consensus meetings. 312 women (38%) were recalled for further assessment. 40 cancers were detected in these women. Invasive ductal carcinoma was the most dominant type of breast cancer accounting for 16 cases. The most common mammographic feature resulting in discordance was asymmetric density.

**Conclusion:** Consensus arbitration is proving to detect a range of cancer types with invasive ductal carcinoma being the most common and asymmetric density the most common mammographic feature. Cancers detected through arbitrations are beneficial for film readers to review, highlighting subtle cancers and reading traits. Interval cancer detection rates for the unit are consistently within limits of national standards, highlighting optimum cancer detection through three yearly breast screening.

## P24. Axillary staging in breast cancer patients: Role of Breast MRI and second look axillary Ultrasound (US)

### **Dr Farah Sadrudin**^1^, Dr Maria Carrillo^1^, Dr Felicity Jenkins^1^, Ben Wild^1^, Dr Marianna Telesca^1^

#### ^1^Worcestershire Acute Hospitals NHS Trust, Worcester, United Kingdom

*Breast Cancer Research* 2024, **26(1)**:P24

**Background:** Axillary node metastases are an important predictor of overall recurrence and survival in breast cancer patients^1^. US is the main method for axillary node evaluation^2^. However, breast MRI has superior visualisation of the global axilla regardless of body habitus^2^.

**Aim:** To evaluate the accuracy of breast MRI and second look axillary US in identifying pathological lymph nodes.

**Methodology:** All patients who underwent axilla US after breast MRI between 01/01/2020–07/08/2022 were identified. Cases included: second look US due to abnormal MRI, those where axillary assessment had not been performed initially with US, and where planned biopsy was indicated after MRI. Using electronic health records, MRI, US and pathology reports were reviewed.

**Results:** Out of 56 cases, 41 demonstrated abnormal axillary nodes on MRI, compared to 26/56 on initial US, and 37/56 on US after MRI. A total of 21/56 cases had pathology positive nodes. 76% (16/21) were demonstrated on MRI, 48% (10/21) on initial US, and 71% (15/21) on US after MRI. MRI identified 9 cases of axillary metastases that were not identified on the initial US. 39% (16/41) of nodes were pathology proven metastatic out of 41 cases classified as malignant on MRI.

**Conclusion:** This study established that 76% of positive pathology cases were correctly identified on MRI. 9 nodes initially identified as normal on US and abnormal on MRI, were subsequently pathology positive. Breast MRI plays an important role in staging the axilla. Second look axillary US should be considered when MRI appearances suggest possible axillary involvement.

## P25. Double reporting with arbitration in the symptomatic setting: Is it worth it?

### **Laura Sewell**^1^, Dr Kirsty McNeil^1,2^, Dr Sarah Savaridas^1,2^

#### ^1^NHS Tayside, Dundee, United Kingdom; ^2^University of Dundee, Dundee, United Kingdom

*Breast Cancer Research* 2024, **26(1)**:P25

**Background:** At our centre all mammograms, including cold-reporting and one-stop clinics, are double-read with arbitration of discrepancies. We seek to evaluate the impact of this practice and any variation between experienced and inexperienced reporting teams.

**Methods:** Arbitration cases were reviewed over two 12-month periods. Between 20/04/15–19/04/16 there was an experienced team, between 01/10/20–30/09/21 there were more inexperienced readers. Cold reporting and one-stop clinics were considered separately. First, second and arbitration reader opinions, malignant and benign biopsies and follow-up data (minimum 22 months) were recorded.

**Results:** Cohort 1 – experienced team: 4654 mammograms were performed with 36 (0.7%) discrepancies, 25 from cold-reporting, 11 one-stop cases. 12 (48%) of cold-reporting cases were recalled resulting in three cancer diagnoses and 3 benign biopsies. 4 (36%) of one-stop cases were recalled with one cancer diagnosis. On > 7 years follow-up one patient developed cancer close to a benign biopsy site, one died of distant recurrence.

Cohort 2 – less experienced team: 3738 mammograms were performed with 92 (2.5%) arbitrations, 54 from cold-reporting, 38 one-stop cases. 10 (19%) of cold-reporting cases were recalled with no additional cancers identified. 10 (26%) of one-stop cases were recalled with three cancer diagnoses. One cancer was missed and subsequently diagnosed via breast screening.

**Conclusion:** Overall 0.8/1000 additional cancers were detected, with no difference between the cohorts. More additional cancers were detected in clinic patients with the inexperienced team. Less than half of discrepant cases were recalled through arbitration. The benefit of double reporting is minimal, arbitration markedly reduces unnecessary recalls.

## P26. A Prospective Analysis of Screen Detected Cancers Recalled and Not Recalled by Artificial Intelligence in an NHS Breast Screening Service

### **Samantha Smith**^2^, Katie Walker-Stabeler^1^, Dr Michael Siafakas^1^, Dr Sally Bradley^1^

#### ^1^University Hospitals Birmingham NHS F Trust, Birmingham, United Kingdom; ^2^University Hospital Coventry and Warwickshire

*Breast Cancer Research* 2024, **26(1)**:P26

**Background:** Analysis of artificial intelligence demonstrates great potential to perform as an independent reader in a screening double reading process and non-inferiority to human reader on screening key performance indicators.

South Birmingham Breast Screening Service has undertaken a service evaluation of Mammography Intelligent Assessment (MIA) software from Kheiron Medical Technologies.

**Method:** MIA ran as a prospective silent single reader in parallel to the standard double reading for 8 months. MIA gave a binary recall/no recall decision with regions of interest which were reviewed.

The positive concordant and positive and negative discordant cases were reviewed.

We recorded the incident/prevalent screen, arbitration rate, individual’s age, breast density, laterality of any abnormality, mammographic features (type of abnormality, size, site, uni/multi focality), mammogram machine manufacturer, biopsy method and all pathology results.

**Results:** There were 416 recalls to assessment by the human readers, 78 were diagnosed with breast cancer and of these 20 had not been recalled by MIA. There were 270 that MIA did not recall to assessment but human readers did and of these 20 had cancer.

There were 146 positive concordant cases of which, 58 were found to have breast cancer. Of the 604 negative discordant cases, 6 were recalled and 2 cancers were diagnosed in the relevant breast quadrant.

**Conclusion:** Artificial Intelligence shows great potential in breast imaging at a time when the NHS is under great pressure.

This work demonstrates that AI missed and found cancers with a variety of mammographic features, sizes and pathologies.

## P27. A Step-by-Step Guide for Reporting DIEP Computed Tomography Angiograms in Autologous Breast Reconstructions

### **Dr Shahana Sulaiman**^1^, Dr Nimmi Nargheese Thazhathe Peedika^2^, Dr Thaj Rehman^1^

#### ^1^Mid and South Essex NHS Trust, United Kingdom; ^2^University Hospitals Birmingham NHS FT, Birmingham, United Kingdom

*Breast Cancer Research* 2024, **26(1)**:P27

The use of Deep Inferior Epigastric Perforator (DIEP) flap in autologous breast reconstruction offers numerous advantages, such as improved patient satisfaction, reduced risks, favorable donor site morbidity, shorter hospital stays, and superior cosmetic outcomes. This paper delineates a systematic approach for reporting donor site DIEP Computed Tomography Angiography (CTA) before undertaking autologous breast reconstruction surgery.

Literature review underscores the advantages of CTA, including reduced flap harvest time and complications. CTA significantly mitigates partial and total flap loss compared to Doppler assessment alone. Despite advancements in Magnetic Resonance Angiography (MRA), CTA still provides superior images and improved perforator detection.

The DIEP CTA technique incorporates supine patient positioning, contrast material administration, and scanning from the pubis to above the umbilicus. Various imaging projections, such as axial and coronal Maximum Intensity Projections (MIP), Multi-Planar Reconstructions (MPR), 3D MPR, and surface marking, are instrumental in the evaluation of the reconstruction process.

Systematic reporting of DIEP CTA involves several key steps: identification of DIEA types, estimating flap volume and weight, location of the dominant DIEP on the axial plane, determination of perforator location from a reference point, identification of the perforasome, assessment of the superficial inferior epigastric size and distribution, documentation of venous anatomy, and notation of any incidental anomalies.

By implementing this succinct and systematic approach, the precision and efficiency of autologous breast reconstruction can be significantly enhanced. Utilization of CTA imaging and adherence to the outlined reporting methodology will enable surgeons to optimize patient outcomes and ensure the success of autologous breast reconstruction.

## P28. MRI characteristics predicting recurrence/ metastases in breast cancer patients receiving neoadjuvant chemotherapy

### **Dr Aisha Syed**^1^, Dr Jyoti Bansal^1^, Megan Wallace^2^

#### ^1^University Hospital Wales, Cardiff, United Kingdom; ^2^Cardiff University, Cardiff, United Kingdom

*Breast Cancer Research* 2024, **26(1)**:P28

**Aim:** To evaluate MRI and tumour characteristics predicting recurrence or metastases in breast cancer patients post Neoadjuvant chemotherapy (NACT).

**Methods:** A retrospective evaluation of all Breast MRIs for NACT monitoring between 2009 and 2018 was performed. All patients were followed up for at least five years. Factors including patients’ age, tumour size, receptor status, number of lymph nodes, and MRI characteristics were evaluated.SPSS (ver 27) was used for statistical evaluation, and p < 0.05 was considered a significant result. Binomial logistic regression was used to evaluate factors controlling for other variables. The median age of patients was 45 years (range 25 to 73).

**Results:** Out of 135 patients, 114 had adequate data for evaluation in this study. Thirty-three (28.9%) patients showed local recurrence or metastases. The median time to an event from the date of diagnosis was 35 months (range 0–144 months). Compared to a non-mass enhancement, a mass-like enhancement was statistically associated with fewer events (p = 0.011). The factors most significantly associated with an event were triple negative (TN) status, a higher number of lymph nodes on baseline MRI and post-surgery (ypN). No significant association was found between T stage, tumour grade or MR response pattern (concentric versus crumbling).

**Conclusion:** Of all breast cancer patients receiving NACT, 28.9% showed an adverse event at a median of 35 months. Factors predicting an event in breast cancer patients receiving NACT were TN receptor status, non-mass enhancement on MRI and higher lymph node status.

## P29. The Accuracy of MRI in detecting the presence of residual disease post Neoadjuvant Chemotherapy: The Welsh Experience

### **Dr Megan Wallace**^2^, Dr Aisha Syed^1^, Dr Jyoti Bansal^1^

#### ^1^University Hospital Wales, Cardiff, United Kingdom; ^2^South Wales Deanery, United Kingdom

*Breast Cancer Research* 2024, **26(1)**:P29

**Aim:** To evaluate the accuracy of MRI in detecting the presence of residual disease in breast cancer patients post Neoadjuvant chemotherapy (NACT) in our current practice and compare it with published literature.

**Methods:** A retrospective evaluation of all Breast MRIs for NACT monitoring between 2009 and 2018 was performed. All patients were followed up for at least five years with the median age of patients at 48 years (range 23 to 73). Post NACT MRI scan reports were compared with histological findings to determine the accuracy with published data.

**Results:** Out of 135 patients, 101 had adequate data for evaluation in this study. 76 patients (75%) had an accurate MRI prediction of response to NACT reported. Out the patients that had inaccurate prediction of response to treatment 68% were triple negative and the remaining 32% were HER 2 positive. (34%) still had residual disease detected on resection of the tumour despite MRI reporting response to NACT as complete. 57 patients (76%) whose MRI showed partial response had partial histological response. 20% of MRI reports which showed residual disease in breast cancer patients had complete response to NACT demonstrated upon histology findings.

**Conclusion:** For detecting residual disease post NACT, MRI was found to have a sensitivity of 90.4% a specificity of 63% a PPV of 83.5% and NPV of 75.7%. This data is in line with the available publish data. Of those with inaccurate MRI findings. MRI findings predictions were least accurate in triple negative breast cancer.

## P30. Identification of incidental breast cancers by Radiologists: results of a full cycle audit

### Dr Claire Filippini^1^, **Dr Louise Wilkinson**

#### ^1^Oxford University Hospitals NHS Foundation Trust, United Kingdom

*Breast Cancer Research* 2024, **26(1)**:P30

Breast cancer (BC) is the most common UK cancer type, with 1 in 8 women diagnosed during their lifetime. Screening for BC automatically stops after 70 years, unless women continue to self refer, but the risk of BC continues to increase with age.

Early diagnosis increases the chance of recovery, and therefore it is vital that radiologists are aware of the early signs of BC and look out for these when reporting imaging performed for another reason, such as a CT pulmonary angiogram (CTPA).

Cycle one of this audit was performed in 2021; 892 women were identified who had been diagnosed with BC between 2011 and 2021; of these, 123 had had a CT scan which included the breasts available prior to their diagnosis, in which a BC had been visible and there was the opportunity for a radiologist to report it. Only 49% of visible cancers were reported by radiologists. The results were reported to the local radiology team and the audit repeated in 2023. Data was analyzed for 1000 women diagnosed with BC since 2021 and whom had had a CT performed prior to diagnosis and after the results of the initial audit were delivered. In this cycle, 57 women had had an incidental BC visible on CT, and these were reported in 81% of cases. Therefore, there has been a significant improvement in radiologist identification of incidental breast cancers between the 1st and 2nd audit cycles (49% and 81% respectively), which positively impacts cancer detection pathways.

